# Bladder Cancer: A Simple Model Becomes Complex

**DOI:** 10.2174/138920212801619232

**Published:** 2012-08

**Authors:** Giovanni Battista Di Pierro, Caterina Gulia, Cristiano Cristini, Giorgio Fraietta, Lorenzo Marini, Pietro Grande, Vincenzo Gentile, Roberto Piergentili

**Affiliations:** 1 Dipartimento di Scienze Ginecologico-Ostetriche e Scienze Urologiche, Policlinico Umberto I, Sapienza - Università di Roma; 2 Dipartimento di Chirurgia d’Urgenza, Azienda Ospedaliera S. Andrea, Sapienza - Università di Roma; 3 Dipartimento di Biologia e Biotecnologie, Sapienza - Università di Roma

**Keywords:** Bladder carcinoma, urinary tract, NMIBC, MICB, carcinoma *in situ*, CIS, FGFR3, TP53, epigenetics, small non-coding RNA, environmental causes of bladder carcinoma.

## Abstract

Bladder cancer is one of the most frequent malignancies in developed countries and it is also characterized by a high number of recurrences. Despite this, several authors in the past reported that only two altered molecular pathways may genetically explain all cases of bladder cancer: one involving the *FGFR3* gene, and the other involving the *TP53* gene. Mutations in any of these two genes are usually predictive of the malignancy final outcome. This cancer may also be further classified as low-grade tumors, which is always papillary and in most cases superficial, and high-grade tumors, not necessarily papillary and often invasive. This simple way of considering this pathology has strongly changed in the last few years, with the development of genome-wide studies on expression profiling and the discovery of small non-coding RNA affecting gene expression. An easy search in the OMIM (On-line Mendelian Inheritance in Man) database using “bladder cancer” as a query reveals that genes in some way connected to this pathology are approximately 150, and some authors report that altered gene expression (up- or down-regulation) in this disease may involve up to 500 coding sequences for low-grade tumors and up to 2300 for high-grade tumors. In many clinical cases, mutations inside the coding sequences of the above mentioned two genes were not found, but their expression changed; this indicates that also epigenetic modifications may play an important role in its development. Indeed, several reports were published about genome-wide methylation in these neoplastic tissues, and an increasing number of small non-coding RNA are either up- or down-regulated in bladder cancer, indicating that impaired gene expression may also pass through these metabolic pathways. Taken together, these data reveal that bladder cancer is far to be considered a simple model of malignancy. In the present review, we summarize recent progress in the genome-wide analysis of bladder cancer, and analyse non-genetic, genetic and epigenetic factors causing extensive gene mis-regulation in malignant cells.

## INTRODUCTION

### Epidemiology of Bladder Carcinoma

Bladder carcinoma (BC) is the most common malignancy of the urinary tract [1] and one of the most frequent cancers worldwide. According to the US National Cancer Institute (NCI) website (accession: June 2012) (http://www.cancer.gov/), cases diagnosed in 2005-2009 from 18 SEER (Surveillance, Epidemiology and End Results) geographic areas are 37.0 per 100,000 men and 8.9 per 100,000 women, with a men:women ratio of approximately 4:1. Estimated cases from NCI for year 2012 show that, among cancers, BC represents the sixth cause of disease and the second urological malignancy after prostate cancer; moreover, BC is the most common urological tumor in China [2]. Recurrence with metastasis is a frequent cause of death, with some studies highlighting this phenomenon in up to 70% of patients [3]. Indeed, overall tumor-specific 5-year survival rate for years 2002-2008 is around 77%, but it is only 5.5% after cancer has metastasized (NCI, June 2012). This also means that BC is associated to the highest costs in patients surveillance compared with other tumors; according to available data (http://progressreport.cancer.gov/) approximately 3.5 billion dollars per year are spent in the US on bladder cancer treatment.

### Non-Genetic Factors Affecting the Epidemiology of Bladder Carcinoma

Active and passive tobacco smoking is the most well established risk factor for BC in both sexes [4, 5]. There is a direct relationship between incidence of BC and the exposure to smoke, in both duration (years since starting) and quantity (cigarettes per day); BC incidence is also higher in people who started smoking at a younger age or are exposed to environmental tobacco smoke during childhood [6, 7]. The second most important risk factor for bladder cancer is occupational exposure [7], a problem known since at least year 1895 [8] and related to 20-25% of all bladder cancer cases.

Dietary factors are connected to BC formation and/or development. It is established that this tumor is directly related to chronic exposure to arsenic, such as that present in drinking water. This is evident in places in which Blackfoot disease (a typical illness related to high arsenic levels in blood) is endemic, as in Taiwan [9], Japan [10], Argentina [11], Chile [12] and the New Hampshire in the United States [13]. Other trace chemical elements were evaluated in literature, and it has been shown that the increase of copper and the decrease of iron and zinc may be involved in BC occurrence [14]. Also the anti-oxidant role of selenium has been studied [15]; interestingly, the role of Vitamin E has been demonstrated as well [16], indicating that anti-oxidants are powerful tools in BC prevention. Although some scientific reports seemed to confirm this, recent data exclude any relationship between BC and alcohol drinking [17, 18]. This result is particularly interesting and somehow surprising, since a relationship between alcohol and BC would be plausible, due to (i) the causal association between alcohol drinking and several types of cancer [19-22], and (ii) the presence inside the bladder and urine of acetaldehyde [23], a metabolite of alcohol and a known carcinogenic compound in humans [20, 24]. These results are applicable considering either the quantity or the different types of alcoholic beverages [18, 25]. Instead, milk consumption seems to show an inverse relationship with BC [26], another unexpected result considering that high milk intake is a risk factor for both ovarian [27] and prostate [28] cancers, two tumors affecting the urogenital system. In the light of earlier studies on milk components, the Authors suggest that the effects of milk may reside in the fats it contains. A recent meta analysis suggests that it is also possible that no relationship at all exists between milk intake and BC development [29], thus this topic is still controversial [30].

BC, and especially the invasive squamous cell carcinoma (SCC), is directly related to the presence of chronic urinary tract infection. Bladder schistosomiasis (bilharzia) is a very common parasitic infection with about 600 million people exposed to infection. This pathology has been considered a definitive cause of urinary bladder cancer with an associated five-fold risk [31]. Actually, it is acknowledged that molecules mediating immune response are also involved in BC development, such as interleukin-2 (IL-2) [32] and, possibly, IL-4, IL-4R and IL-13 [33]. Moreover, in mice it is has been recently shown a relationship among induced inflammation, miR and TP53 expression [34], all of these being involved in BC incidence (see next sections).

External beam radiation therapy (EBRT) for urogenital malignancies increases the rate of secondary bladder malignancies: standardized incidence ratios for bladder cancer developing after radical prostatectomy (RP), EBRT, brachytherapy (BT), and EBRT-BT were significantly higher than that of the general United States population. Thus, the increased risk of bladder cancer in patients undergoing ERBT, BT or ERBT-BT should be taken into account during follow-up, particularly for young patients [35]. Age is also important for survival after radical cystectomy (RC): it has been demonstrated that greater age is associated with adverse outcome [36]. As for chemotherapy, the use of cyclophosphamide, an alkylating agent used for treatment of lymphoproliferative diseases and other non-neoplastic diseases, has been correlated with later development of muscle-invasive bladder cancer (MIBC), with 6-13 years of latency. Similarly, acrolein, a metabolite of cyclophosphamide, is responsible for the increase in the incidence of BC and this effect occurs independently of the association of hemorrhagic cystitis with the same treatment [37, 38].

BC differently affects the two sexes. As stated before, males are more prone to BC than females (4:1 ratio); however, after RC of urothelial cell carcinoma (UCC), the prognosis of female patients is worse than that of male ones [39, 40-42]. Interestingly, survival of females after any cancer treatment is always better than that of males [43] except for BC. Social and cultural parameters may be taken into account to better explain survival of females to general cancers, such as a major awareness of women for their health, a minor exposure to risk factors, a lower age-related mortality interacting with cancer-specific survival. Thus, in this perspective, BC is a tumor behaving differently from the others. Moreover, this is particularly evident if the comparison is made according to cancer stage (Table **1**): extrapolation of these data reveals that BC staging T1 to T3 affects both sexes equally as for survival, but a difference is evident for stage T4a [42]. Explanations of these data are still missing, although some environmental parameters (presence of specific carcinogens, anatomical and/or hormonal differences) are discussed in literature [44, 45].

Socio-economic explanations may be also taken into account when discussing ethnic differences in BC survival. Black people are known to have poorer survival to BC compared to whites, Hispanics and Asian/Pacific Islanders in the United States, especially because blacks show higher BC stage at presentation [46, 47]. However, differences in survival are present even if patients show similar BC stage, grade and treatment [48]. Some Authors [47] conclude that these disparities may be due, at least partly, to disparity in quality of treatment, access to and quality of care, surveillance after primary treatment, and comorbidity. Even marital status might be taken into account, when evaluating cancer survival [49].

### Diagnosis, Prognosis and Treatment of Bladder Carcinoma

BC patients at time of presentation may be broadly subdivided into two groups: those having a non-muscle-invasive bladder cancer (NMIBC) staged Ta and T1, and those with a muscle-invasive disease (MIBC), staged T2 to T4 (Table **1**) [50, 51]. The former group represents approximately the 75% of the patients, and the latter the remaining 25%. Among patients with NMIBC, 60% are expected to show recurrence of the disease and 1 out of 5 patients will develop a MIBC [52]. Survival rates after 5 years for NMIBC patients is around 88-98%; instead, as for the MIBC patients, about half of them are expected to die within 5 years [53], mainly because of metastases [54] (Table **2**). Risk factors associated to progression to MIBC include deeper invasion of the *lamina propria*, tumor grade and size, concurrent presence of carcinoma *in situ* (CIS), tumor multiplicity, and recurrence of NMIBC [55]. Tumor types of the BC are urothelial cell carcinoma (UCC, previously called transitional cell carcinoma, TCC), squamous cell carcinoma (SCC), adenocarcinoma, and other sporadic lesions [56]. UCC begins in the cells lining the inner-most tissue layer of the bladder; SCC arises from the squamous cells of the bladder epithelium and it is frequently associated to long-term infection or irritation of this layer; finally, adenocarcinomas usually arise from bladder secretory cells, frequently of urachal origin. At diagnosis, 90% of all BC are UCC, and three fourths of them are papillary tumors localized in the urothelium or in the *lamina propria*. Less than 8% are classified as SCC and 2% are adenocarcinomas. Recurrence is the main problem for NMIBC patients, since up to 80% of them may relapse; instead, progression is the main problem for patients in T1 stage and CIS [52, 57, 58]. For this type of lesion a ‘risk calculator’ had been created [52], which is available online in the European Organisation for Research and Treatment of Cancer (EORTC) web site (http://www.eortc.org/). For MIBC patients, the main predictors of outcome are lymph node involvement, tumor stage and grade, lymphovascular invasion and histological subtype as for the tumor features; time from diagnosis to surgery, patient age and gender as for clinical factors [59].

Diagnosis of NMIBC (Ta/T1 grade BC plus the flat, high-grade CIS tumors confined to the mucosa) may be performed by urine cytology, ultrasonography, and cystoscopy with description of the tumor (site, size, number and appearance) and mucosal abnormalities. However, a complete and correct transurethral resection (TUR) is essential to make a correct diagnosis and remove all visible lesions [50]. Instead, the standard treatment for patients with MIBC (T2-T4a, N0-Nx, M0) is radical cystectomy (RC) with lymphadenectomy [51]. However, this ‘gold standard’ only provides 5-year survival in about 50% of patients [60]. In order to improve these unsatisfactory results, the use of peri-operative chemotherapy has been introduced. Some studies demonstrate that neoadjuvant cisplatin-containing combination chemotherapy improves overall survival by 5-7% at 5 years and should be considered in MIBC, irrespective of definitive treatment [61, 62], but its use is still a matter of debate [63]. Patients with immobile tumors (stage T4b) will receive chemotherapy or radiotherapy, occasionally followed by salvage cystectomy [53].

To date, recurrence of BC is explained by two different theories: the field-cancerization hypothesis, and the intraluminal seeding and implantation hypothesis. The first theory hypothesizes that multi-focal tumors are a consequence of carcinogen exposure of the entire urothelial layer [64, 65]. The second theory, supported by molecular data, suggests that multi-focal tumors are a consequence of clonal evolution from a single transformed cell [66, 67]. Notably, a recent paper demonstrates that the normal mucosa of human bladder contains multiple stem cells, each responsible for the turnover of the surrounding cells during bladder layers renewals, and that any clone may be replaced by surrounding clones [68].

## THE GENETICS OF BLADDER CARCINOMA

### Genes Controlling Cell Cycle Regulation

Several Authors report that BC is an example of human malignancy where molecular profiling can be restricted to the analysis of the function of only two genes, *FGFR3* and *TP53* [69-72]. However, in the last years several data show that this vision of the problem may be limited (Table **3**). Several functions are necessary for the maintenance of cell homoeostasis, and alterations in genome stability, cell cycle control, apoptosis, angiogenesis, cell signalling and hormone receptor functions may all drive to neoplastic transformation and promote tissue invasion. The tumor suppressor gene *TP53* is the most commonly mutated gene in cancers, including MIBC [73], since it plays essential roles in the regulation of cell proliferation, apoptosis and inhibition of angiogenesis [74]. Many studies demonstrate that altered function of this gene, as well as that of its relative *TP63* [75], either because of a mutation in the coding sequence or for alterations in its regulation, may be predictive of a poor outcome in both NMIBC and MIBC; moreover, the levels of the TP53 protein increase in normal urothelium to NMIBC to CIS to MIBC to metastatic BC [76-78]. However, some scientific reports discourage the use of *TP53* status alone in BC as a final outcome predictor [73, 79, 80]. TP53 controls the proper function of TP21, another key protein in cell cycle regulation. TP21 [81], together with TP27, is an inhibitor of the G1/S cell cycle transition thanks to its ability to regulate cyclin dependent kinases cdk2/4 [82, 83]. Thus, also these two proteins are good markers of the BC final outcome [78, 83-85]. Indeed, TP27 regulation is under control of Nkx.28, an NK-2 homeobox containing protein that also regulates cyclin D1, FOXO3a (a transcription factor) and the MEK/ERK pathway [86]. The contemporary malfunction of TP53 and TP21 is associated to higher relapse rate and worse survival after RC, compared to TP53 alterations only [87]. Instead, TP27 is a good predictor of outcome for MIBC, but not for NMIBC [76, 77]. As a consequence of the role of TP21/TP27, also cyclins are potentially good markers of BC progression. The primary protein in this stage, with importance in BC, is cyclin E1, whose decreased expression is frequently associated with advanced, metastatic disease [88]. Another cell cycle regulator involved in BC development is the Retinoblastoma (Rb) protein. Rb plays a role in stem cell maintenance, replication and differentiation, by controlling the G1/S transition; nonetheless, its ability to become a molecular predictor of BC outcome seems limited [89]. Finally, it has been recently demonstrated that also Polo-like kinase 1 (Plk1) has a significantly higher expression in BC and it is positively correlated with its grade, stage, recurrence, and metastasic invasion [90].

### Genes Controlling Apoptosis

Even if cell cycle progression is somehow deregulated, the cell may still remain under control thanks to the activation of the apoptotic pathways; indeed, their inactivation is frequent in all cancers and, as expected, also in BC [91]. Among the others, we can here remember the role of the protease Caspase-3, frequently associated to high grade BC [79, 92, 93]; Fas, a death receptor which is down-regulated in many BC samples [94, 95]; Bcl-2, an anti-apoptotic protein which is up-regulated in one third of the RC specimens and that is able to promote chemotherapy resistance in neoplastic cells [92]; and Survivin, another inhibitor of apoptosis acting on caspases and promoting tumor cell invasion; Survivin is up-regulated in BC, especially in high grade tumors [96-98]. Another good candidate as a molecular BC marker in this group is cellular inhibitor of apoptosis protein 1 (cIAP1), belonging to the same protein family (IAPs) of Survivin; cIAP1 is a nuclear shuttling protein able to block both the intrinsic and the extrinsic apoptotic pathways by inhibiting caspases activity; indeed, cIAP1 over-expression is highly correlated with BC recurrence, progression, resistance to chemotherapy and poor prognosis in MIBC [99]. Finally, we remember the FLICE-inhibitory protein (c-FLIP) long splicing form, which has a pivotal role in TRAIL- and CD95L-mediated apoptosis resistance [100], and the TRAIL protein itself that, together with its death receptors DR4 and DR5, is a good prognostic marker for BC [101].

### Genes Controlling Angiogenesis

Solid malignancies need food and oxygen supplies to self-maintain. It is known, for example, that a high value of microvessel density is a potential predictor of poor prognosis in BC and that this parameter is also associated with lymph node metastasis formation [102]. Thus, also genes involved in angiogenesis play an important role in BC. Beyond the already cited TP53, proteins of the vascular endothelial growth factor (VEGF) family promote cell replication and migration, and are up-regulated in BC specimens, especially those of high grade [79]; VEGFs influence metastasis development and, consequently, prognosis [103, 104]. Similarly, the basic fibroblast growth factor (bFGF), with pro-angiogenic activity, is present in urine samples from BC patients, but it is absent in controls [105]; moreover, its amount increases with more advanced stages of the disease [102]. Thrombospondin-1 is an important component of the extracellular matrix and a potent inhibitor of angiogenesis, and is involved in BC progression as well [102]; however, its use as a prognostic marker heavily relies on the concurrent expression of TP53 [102] since the expression of these two proteins is strongly linked in BC development.

### Genes Controlling Cell-Cell Interactions and Signalling

The neoplastic mass, established and supplied with nutrients, having lost internal cell cycle control and escaped intrinsic/extrinsic induction of apoptosis, must face interactions with surrounding, likely normal, tissues and organs. Uroplakins [106] are integral membrane proteins specific of the urotelium; they are expressed in approximately 50% of MIBC, but not in other tumors and they are absent in the more aggressive stages of MIBC [107]. Also signalling pathways are of great importance for cancer ‘survival’ inside the body; in this perspective, BC is not different from other cancers. Proteins like those of the epidermal growth factor receptor (EGFR) family are indeed involved in poor prognosis. For HER2, results published so far are puzzling, since some studies emphasize a correlation between its high expression and poor prognosis [108, 109] while other studies do not [110, 111]; a similar situation occurs for other members of this protein family [112, 113]. Instead, the implication of the RAS proteins [114] and of their effectors (such as RIN1 [115] or BNIP3 [116], both effectors of HRAS) in BC is widely accepted: approximately 13% of specimens has a mutation in one of the components of the RAS gene family (HRAS, NRAS, KRAS2) and one report shows that RAS and FGFR3 (see below) mutations are mutually exclusive in BC [117]. The role of RAS in activating mitosis (interaction with MAPK and PI3K) also suggests a role for these two proteins in BC. Indeed, PTEN and PIK3CA are both involved in BC, and are both part of the phosphatydilinositol 3-kinase (PI3K) pathway [118, 119]. Downstream of PI3K (improper) activation in BC, the phosphorilation of AKT promotes mTOR pathway activation [120], a typical feature of this cancer. In this pathway, inactivating mutations of tuberous sclerosis protein 1 (TSC1) may play similar roles in it. Annexin family is a group of twelve proteins playing a role in several processes, including tissue growth; among them, Annexin10 (ANXA10) had been validated as a good marker for the analysis of the aggressiveness of BC [121, 122]. Moreover, ANXA10 and TP53 act synergistically (inverse correlation) towards high grade/stage NMIBC to MIBC progression, likely because down-regulation on ANXA10 promotes cell migration [122]. Another gene related to cell migration is longevity assurance homologue 2 of yeast LAG1 (LASS2), which is a tumor metastasis suppressor gene; a recent report shows that this gene is down-regulated in BC and it is associated to poor clinical prognosis [123]. Other worth mentioning proteins involved in cytoskeleton homoeostasis and BC invasiveness are Cortactin [124] and the actin binding protein Profilin-1 [125]. ADAM28 [126], ADAM12 and ADAM17 [127] are metalloproteinases, members of a family of membrane-anchored glycoproteins with adhesive properties, that are frequently expressed in several types of tumors; among them, ADAM28 is considered another good molecular marker of BC [126]. To date, the role of androgen, oestrogen and progesterone receptors in BC are a debated topic [127]; however, recent scientific reports had been published indicating that the estrogen receptor beta may be a prognostic marker of recurrence-free rate in NMIBC, potentially through suppression of the cadherin switch [128, 129]. Also androgen receptor and oestrogen receptor alpha may be deregulated in BC [129]. Finally, other possible candidate proteins as BC molecular markers have been reported in a recent proteomic analysis [130], the most promising of which are apolipoprotein E (APOE), leucine-rich alpha-2-glycoprotein (LRG1) and, above all, fibrinogen beta chain precursor (FGB) and alpha-1 antitrypsin (SERPINA1).

Among the signalling proteins, one of the best characterized in BC is the fibroblast growth factor receptor 3 (FGFR3) [131, 132]. This protein is involved in several types of diseases and cancers, such as skeletal dysplasias (achondroplasia, thanatophoric dysplasia), skin disorders and cancer (epidermal nevi, seborrheic keratosis, acanthosis nigricans, myeloma, seminoma, bladder and cervical carcinoma), cartilage growth anomalies (inhibition of chondrocyte proliferation) [133]. FGFR3 is a tyrosine kinase receptor implicated in cell cycle control and angiogenesis. Improper activation of this gene (by mutations in the coding sequence or by alterations in its regulatory pathways) is present in 60% of BC samples, as underlined for example in studies of tissue microarrays [134, 135]; indeed, FGFR3 improper function is a typical feature of NMIBC [136]. FGFR3 direct and indirect interactors include MAPK, phospholipase Cγ, protein kinase C, Src, PI3K, AKT [137, 138] and STAT1 (leading to TP21 accumulation) [133, 139].

### Genes Involved in Other Cellular Functions Important for BC Development

None of the aforementioned genetic alterations, taken alone, is sufficient to predict the outcome of the disease, nor simply to assess the stage and/or grade of the BC. Cancer is a multi-step disease, thus several genes must be mutated or deregulated in a cell to promote its neoplastic transformation. Thus, also genes controlling genome integrity (such as those involved in DNA replication or repair) are of crucial importance because of their “mutagenic” effect. Genes like that coding for the high-mobility group box 1 protein (HMGB1, having intranuclear and extracellular functions and involved in DNA transcription and repair) [140], methylene tethrahydrofolate reductase (MTHFR, an enzyme necessary for nucleotide biosynthesis) [141], the single strand DNA damage repair proteins hOGG1 (a glycosylase involved in base excision repair), XPD (one of the proteins affected in Xeroderma pigmentosum disease) [142] and ERCC1 (involved in nucleotide excision repair) [143], and the double-stranded DNA breaks repair proteins ERCC2, NBS1, XPC [144], XRCC3 [145] and PARP1 [146], are all involved in the formation/development of BC.

The alteration of the function of detoxifying proteins [147] such as UDP-glucuronosyltransferases (UGTs), which facilitate cellular removal of bioactivated forms of aromatic amines found in tobacco smoke and industrial chemicals [148], or glutathione S-transferases (GSTT1, GSTM1, GSTP1) [149, 150] and CYP1B1 (a member of the cytochrome P450 superfamily of enzymes) [151], all implicated in the inactivation of procarcinogens, are likely causes of BC susceptibility, by increasing the number of (eventually unrepaired) DNA lesions.

Of course, the more genes are analyzed, the more precise the diagnosis is; however, using mathematical approaches, it has been demonstrated that only a “few” genes – as low as eleven [266] or even less [153] – may be actually analysed to have a sufficiently accurate diagnosis.

### Innovative Approaches for the Identification of New Genes Involved in BC

Recently, new approaches were developed to “indirectly” identify genes involved in BC. One method is the identification of peptides inside urine, to discover patients with MIBC using a minimally invasive technique. The approach is promising, however the reported panel of peptides may be not highly specific [152, 153]. A second approach takes advantage of the *metabonomics*, i.e., the investigation of the presence of metabolites potentially useful as cancer biomarkers and not directly related to any genotype, expression profile, or even proteome [154, 155]. Using this approach, Huang and co-workers [156] were able to demonstrate that carnitine C9:1 and component I may be used together to discriminate, with high specificity and sensitivity, patients with BC, especially those with a low grade disease; however, why these molecules are particularly abundant in these patients is not yet understood. Other twelve potential markers were also identified, which need further validation or have lower specificity/sensitivity. Identifying the proteins (and, consequently, the corresponding genes) responsible for the alteration in the total amount of these metabolites may shed light on other, still unknown, metabolic malfunctions leading to neoplastic transformation in BC.

## EPIGENETIC CHANGES IN BLADDER CARCINOMA: TRANSCRIPTIONAL CONTROL

### Genetic Up- Or Down-Regulation Occurs in BC Cells

Genetic changes in gene expression and/or function are inheritable, frequently irreversible and due to permanent modifications of DNA, either because of small nucleotide sequence changes (point mutations) or because of gross chromosomal rearrangements (translocations, deletions, duplications and its correlated copy-number variations that may quantitatively increase gene expression). Indeed, chromosome 9 total and/or partial loss [157] is associated to BC recurrence, and at least four regions of this chromosome seem crucial in this phenomenon [158]. Epigenetic changes are similarly inheritable, but they are potentially reversible and they are not mediated by alterations in DNA sequence or chromosome structure; instead, they often involve changes in interphase chromatin architecture and, consequently, function. Well known epigenetic gene control mechanisms involve histone modifications (which influence gene expression at the transcriptional level), modification of the DNA itself, such as methylation (that acts at the transcriptional level as well), or even changes mediated by non-coding RNAs (post-transcriptional silencing, see next section). Several epigenetic alterations had been identified in BC in the last years [159, 160].

As for gene over-expression, it is long known its role in BC, although the involved mechanisms are not always clear (gene amplification by copy number variations, chromatin hypo-methylation, small chromosomal rearrangements invisible at the light microscope). Examples of this type involve the (hyper-)activation of (onco)genes such as *c-myc* [161], *E2F3* [162], *Rb* [163], *CCND1* [164], *ID-1* [165] and *AKT1* [166], or the increased level of the highly specific marker BCLA-4 (a transcription factor usually over-expressed in both BC and surrounding benign tissues, but not in normal urothelium [167, 168]). Also prolidase (a metalloproteinase) and its regulator, nitric oxide (NO), may promote angiogenesis and metastasis formation [169]. On the other hand, hypo-activation of key genes is involved in BC development as well, engaging for example *TP53*, *Rb* again [170] and *p16* [171] genes. Direct analysis of mRNA from blood cells allowed the identification of two more mis-regulated genes in BC vs. control cells, namely *C10orf116* (of unknown function) and the *KRT19* keratin-coding gene [172]. Several studies are still running, and the list of altered gene expression is getting longer every week [173].

### The Role of Histone Modification in Chromatin Remodeling and Gene Expression

Histone modifications have been extensively described in BC, at the genomic level, in recent works [174, 175]. This phenomenon may lead to either gene silencing or over-expression; both these phenomena were found in BC [175], and the in-depth study of down-regulated sequences allowed the identification of seven genome regions which are significantly associated with the presence of CIS. On the other hand, those bioptic samples identified for harbouring *FGFR3* mutations, did not display the identified silencing profile. Thus, the silencing phenotype is specifically associated with CIS, high tumor grade and stage, MIBC development and very low frequency of *FGFR3* mutations. Interestingly, some of these seven regions are shared with other cancers, but their number and association is tumor-specific. This means that not only any region itself is necessary yet insufficient for neoplastic transformation, but also that particular combinations of different regions may be distinctive of different types of cancer. Thus, any histone-mediated silenced region specific for BC may not be silenced in another tumor, and vice versa. As expected, alterations in the amount of proteins directly related to histone modification are responsible as well of the BC final prognosis. For example, histone deacetylase 6 (HDAC6) and histone deacetylase SIRT2 are both involved in metastasis formation in BC, mainly (but not only) because they also target the *cortactin* gene, encoding for a cytoskeletal protein [124].

### The Role of Chromatin Remodeling by DNA Methylation

DNA methylation plays an important role in gene silencing and BC development, too [176]. Several genes were found to have an altered expression as a consequence of methylation-mediated gene silencing, especially in the CpG islands surrounding the open reading frames of key genes [177-183]. In the last years, the record of genes epigenetically silenced is growing at high speed. A low level of expression of E-cadherin (a trans-membrane protein involved in cell adhesion) is associated to neoplastic transformation and its lower levels correlate with higher invasiveness and metastatic properties of human cancers, including BC; one of the means of its down-regulation is indeed methylation of the CpG island located at the 5’ end of the gene [184]. Interestingly, in their report [184], the Authors also show that this same methylation may occur spontaneously in elderly individuals. In this perspective, it is worth remembering that hyper-methylation is an aspect of cell reaction to the environment (broad sense), too; not only age, but also gender, smoking history, and cancer development (stage, grade, localization, progression) influence chromatin remodelling [178, 185, 186]. Therefore, both ‘external’ (environment-derived, such as smoke) and ‘internal’ (age, gender) signals of gene silencing may act synergistically to promote cell transformation and BC development. Together with E-cadherin, also the methylation-derived low levels of TP16, TP14, DAPK and RASSF1A may be used to predict BC recurrence and prognosis [187-189]. In particular, RASSF1A [190], together with APC, may be a good prognostic factor for tumor reappearance; moreover, patients with methylated APC or RASSF1A coding genes were also significantly associated with shorter recurrence-free survival [191]. Nonetheless, RASSF1A role as tumor stage and grade marker is still a debated topic [191]. As for *p16*, its role has been demonstrated in BC as a consequence of silencing and/or haplo-insufficiency [171]; remarkably, *p16* silencing may be also a consequence of uropathogenic *E. coli* infection [192]. Also transcription factors are implicated, as expected, in these phenomena. *RUNX3* and *SOX9*, for example, are silenced in BC in at least 70% of the analysed samples [193, 194]; *RUNX3* turned out to be a good candidate for the analysis of BC recurrence and development [195, 196], while *SOX9 *is part of a list of 26 hypermethylated genes in BC-derived tissues [194]. The *Wnt* genes are part of a well studied pathway involved in neoplastic transformation, and their role has been repeatedly demonstrated in BC, especially when the pathway is up-regulated as a consequence of hyper-methylation of its antagonists [197, 198]. For instance, epigenetic silencing of the genes encoding the secreted frizzled receptor proteins (SFRP), antagonists of the WNT pathway, leads to constitutive WNT signalling and promotes the invasive phenotype of tumors [199, 200]. The activin membrane-bound inhibitor (BAMBI) gene is epigenetically silenced in high grade BC and it is correlated to high aggressiveness and invasiveness [201]. It is likely that BAMBI plays its role by fine-tuning the TGF beta signaling pathway [201], which in turn has an essential role in many cellular processes such as cell growth and differentiation, apoptosis and cellular homeostasis. It is important to remember that also growth differentiation factor-9 (GDF-9), which belongs to the TGF beta superfamily of proteins, has been recently identified as a potential oncosuppressor in BC development [202]. Also the role of the *EZH2* gene (which acts as a repressor on various target promoters) had been investigated, showing that EZH2 protein expression and APAF-1 methylation are related to TCC progression and invasiveness [203]. Another hyper-methylated signalling pathway in BC involves the endothelin receptor type B (EDNRB), a G-protein-coupled receptor that activates a phosphatidylinositol-calcium second messenger system. This protein, together with APC (adenomatous polyposis coli), TERT_a and TERT_b (telomerase subunits), are part of a panel of markers able to discriminate between tumors and controls using a minimally invasive technique [204]. Notably, in the same report the Authors also found that the tumor necrosis factor receptor superfamily member 25 (TNFRSF25) coding gene is instead hyper-methylated in controls, but not in BC cells. Conversely, another member of the same superfamily, namely TNFRSF6 (also known as Fas, involved in apoptosis) is down-regulated at both mRNA and protein level in BC cell lines and tissue samples of bladder urothelial carcinoma because of DNA methylation [205]. The same technique, that is the analysis of methylated genes from voided urine, allowed two more groups to identify other genes which are silenced in the same way, (i) *GDF15* (growth differentiation factor 15, involved in apoptosis), *TMEFF2* (tomoregulin-2, important for cell survival), *VIM* (Vimentin, a type III intermediate filament) [206], and (ii) *ZNF154* (a zinc finger protein coding gene), *POU4F2* (a transcription factor), *HOXA9* (a homeobox motif containing protein), *EOMES* (eomesodermin, containing a DNA binding domain) [207]. In particular, the latter work [207] also highlights that BC-derived cells are a mosaic of genomic loci that may be either hypo- or hyper-methylated. Besides the hyper-methylated genes recorded above, there is hypo-methylation, and thus over-expression in malignant cells, of the small proline rich proteins (SRPP) coding genes located on chromosome 1 (SPRR1A/2D/3); of five keratins on chromosome 12 (KRT2A/6B/6C/7/8) (see also [208]); of three keratins on chromosome 17 (KRT10/19/20); of the keratin-associated proteins coding genes on chromosome 21 (KRTAP13-1, KRTAP19-2 and KRTAP20-2); the entire chromosome 21 seems indeed a target of differential methylation in BC cells vs. non malignant cells [207], and this could be related to the “protection” against urological cancers of patients with the Down syndrome [209]. These data fit well with the above-mentioned relationships of histone-mediated silenced/ activated regions; indeed, Down patients are protected against BC but are more prone to leukaemia [210], indicating that the pattern of genomic loci activated/silenced is tumor-specific as suggested before.

The list of methylated genes whose inactivation is related to BC formation and development is increasing every week. Similarly to what described in the previous section, also in this case many cellular processes may be affected. As for intracellular events, we may remember the Myopodin, an actin-binding protein [211]; PMF1, involved in polyamine homeostasis that, in turn, controls cell growth and death [212]; the ARF tumor suppressor [213]; the oncogene TWIST, a transcription factor [214]; several pro-apoptotic proteins such as BCL2, TERT, RASSFIA [215] and DAPK kinase [177, 215, 216]; and last but not least, as expected, FGFR3 [217]. As for extracellular events, i.e., proteins acting mainly in the pericellular matrix, controlling cell adhesion and potentially able to promote metastatic invasion, we may remember TIMP-3, a metalloproteinase-3 inhibitor [213]; Nidogen2 (NID2) [214]; HYAL-1, involved in the degradation of the hyaluronic acid [218]; and collagen type 1α2 [219].

## EPIGENETIC CHANGES IN BC: THE ROLE OF SMALL NON-CODING RNAs IN POST- TRANSCRIPTIONAL SILENCING

### Non-Coding RNAs and BC Development

It is well known that not all transcribed genes encode a protein – for example, tRNA, rRNA, telomerase RNA. In recent years, other non-coding RNAs (ncRNA) have been discovered and involved in numerous mechanisms of gene expression control, mostly silencing. Many of these ncRNA had been identified in BC and several reports indicate their pivotal role in its development (Table **4**). Among the others, the long (>400 nucleotides) non-coding RNA (lncRNA) named *UCA1* has been recently added to the class of ncRNA involved in BC formation [220, 221]. In particular, this lncRNA seems to play a central role in cell cycle regulation by indirectly acting on the PI3K-AKT pathway through the CREB protein deregulation [222].

The small ncRNA (micro ncRNA, also known as miRNA or miR) are a hot topic for the study of their role in cancer [223]. miRNA are usually 22nt long nucleotide sequences which are either transcribed of their own, or they are part of another RNA [224]; they may also be arranged in cluster in some genomic loci, counting more than 50 members [224, 225]. miR structure, indispensable for their function, is an hairpin able to bind target protein-coding mRNA by complementarity. The target is usually one of the ends of an mRNA molecule, but also internal sequences may be used as binding sites; the pairing might not necessarily be a perfect match, indicating that a single ncRNA is potentially able to target several mRNA [226]. Silencing may be obtained either by impeding translation, or by mediating target destruction; however, also up-regulation had been found [226, 227]. Their number in the human genome is now above 1,100 (http://www.microrna.org/microrna/home.do) and still rising. The discovery that miR are involved in neoplastic transformation attracted several scholars, and the number of scientific reports identifying new miR in carcinogenesis is increasing steadily. As expected, both miR [228], and Dicer (one of the proteins responsible for their maturation) [229] are involved in urologic cancers. Interestingly, the role of miR on gene expression depends upon their expression itself, which may be regulated either genetically or epigenetically [225, 230, 231], increasing the level of complexity of this mechanism of gene control. Thus, in some cases it has been found that some miR are down-regulated (thus, likely, this will bring to up-regulation of target mRNA); in other cases, miR are up-regulated, causing the opposite effects [232, 233].

### miR and Their Targets in BC Formation and Development

If we think of up-regulated target mRNA as oncogenes, and down-regulated mRNA as oncosuppressors, the link between miR and BC becomes evident (Table **4**). Indeed, it has been demonstrated that the *FGFR3* mRNA is a target of miR31, miR99a and miR100 [228, 234, 235]; miR21 targets *TP53* [228, 234], *TIMP3* (a metalloproteinase involved in the degradation of the extracellular matrix) and *Bcl-2* (a regulator of apoptosis) mRNA [236], the tumor suppressor genes *PTEN* [237, 238] and *tropomyosin 1* (*TPM1*) [239] and, probably, other predicted mRNA targets involved in BC genesis such as *MSH2* (coding a mismatch DNA repair protein) [240] and the transcription factor *E2F3* [162, 241], which is also a target of miR125b [242]; E2F3 regulation by miR is also dependent upon the Pumilio translational repressor [243]; miR1 induces apoptosis by inhibiting the mRNA of the serine/arginine-rich 9 (SRSF9/SRp30c) protein, important for alternative pre-mRNA splicing of apoptosis-related genes [244]; miR205 is potentially able to target at the same time *TP53*, *PTEN*, *c-erbB-3* (a member of the EGFR family of receptor tyrosine kinases), *cdc42* (a small GTPase of the Rho-subfamily) and *Yes* (a tyrosine kinase coding gene belonging to the *src* family of genes) [245]; miR195, which in other cancer types recognizes different mRNA, in BC seems to identify the cyclin-dependent kinase 4 (CDK-4) mRNA as a preferential target [246]; miR200 family acts on the expression of both *ERRF-1*, a regulator of *EGFR* [247] and of *ZEB1*, a transcriptional repressor of *E-cadherin* [248]; miR200 family may in turn be silenced by TWIST1 (a transcription factor), thus repressing *E-cadherin* gene by up-regulation of *ZEB1* [249, 250]; mir449a acts as a tumor suppressor by down-regulating CDK6, CDC25a (which controls Rb phosphorilation status) and promoting p130 (a Rb-related protein) expression increase [251]; miR493 silences the expression of *FZD4* (a transmembrane receptor) and *RhoC* (a G protein, part of the Ras superfamily of signaling proteins) genes, thus acting itself as an oncosuppressor [252]; miR1826 down-regulates at the same time *CTNNB1* (Wnt/beta-catenin pathway), *MEK1* (Ras pathway) and *VECFG* (a growth factor, part of the Ras pathway) [253]; *ERK5/MAPK7* and *AKT* kinases mRNA are both targets of miR143 [254, 255] which, in turn, if down-regulated, promotes the up-regulation of the proto-oncogene plasminogen activator inhibitor-1 (PAI-1), a common marker of solid tumors [256]; miR221 controls apoptosis through its action on TRAIL [257, 258]; miR129 has two putative targets, namely *GALNT1* (involved in post-transcriptional glycosylation of proteins in the Golgi apparatus) and *SOX4* (a transcription factor) [259]. To further complicate this scenario, it is noteworthy to remember that some of the abovementioned proteins, regulated by miR, are in turn able to regulate miR expression, like TP53, TP63 and TP73 [260]; furthermore, some of the components of the miR maturation machinery are themselves under miR control [260, 261].

### Genome-Wide Approaches Allow the Identification of New miR Involved in BC

Likely, the list of miR targeting genes involved in BC formation will get longer in the next few years. The approach to identify new miR involved in BC is usually a large-scale genetic profiling, where the expression of RNA cellular content is analysed in parallel for several hundreds of genes, in both patients and healthy controls. In this way, in year 2007 it was possible for the first time to identify 10 up-regulated miR in BC [257]. Two years later other studies revealed the presence in BC of both up- and down-regulated miR [259, 262, 263]; then, it was reported that their presence might be revealed also in urine samples of BC patients [264, 265]. In the same years it was also suggested that miR analysis might permit distinguishing high- and low-grade BC; in particular, although with some exceptions, down-regulation may be typical of low-grade BC, while up-regulation is more common in high-grade cancers [234]. Interestingly, also miR expression profile of low-grade BC is different from the high-grade one, indicating that diverse miR are involved in BC and that they may have different effects, likely because they target distinct mRNA [235, 266]. As for regulation, recent data seems to suggest, though, that in BC there is a predominance of miR down-regulation [267, 268], while up-regulation is not common, irrespective of tumor stage and grade [267]. Using genomic approaches, also similarities may be studied: in a recent work [233] the Authors found a number of miR which are deregulated in the same way in three different genitourinary cancers, specifically transitional cell carcinoma (TCC), clear cell renal cell carcinoma (ccRCC) and testicular germ cell tumor (TGCT), indicating that some deregulations are tissue-specific while others are cancer-specific. In more detail: regulation of cell adhesion is impaired in all three tumors, but calcium signalling pathways seem to be specific for TCC and TGCT only; instead, several pathways are deregulated in both TCC and ccRCC, such as TP53 signalling, regulation of cell cycle, actin filaments behaviour and focal adhesion. On the other hand, pathways such as cytokine-cytokine receptors or PPAR signalling are impaired in both TGCT and ccRCC, but not in TCC. Finally, also TCC-specific pathways had been identified; among the others, there are drug metabolism and P450-mediated metabolism, MAPK signalling, chemokine signalling and renin-angiotensin system. This approach also allowed the Authors to subdivide analyzed miR into three clusters: the first, dominated by up-regulated oncogenes; the second, enriched in down-regulated (candidate) tumor suppressor genes; the third, maybe the most interesting, in which the same genes are up-regulated in ccRCC but down-regulated in both TCC and TGCT. This last cluster is probably a “cancer specific” set of genes, while the first two may potentially be the “tissue specific” genes responsible of neoplastic transformation of the genitourinary tract cells.

The situation recently became even more complicated. Recent studies reported that, at least in some cases of BC, it is not the total level of any one miR, but the ratio between two miR that might be important in carcinogenesis. For example, it has been showed that the miR126:miR152 ratio in urine permits the detection of bladder cancer with a sensitivity of 72% and a specificity of 82% [264], while another group explained that the miR21:miR205 expression ratio allows the distinction between invasive and non-invasive BC with a sensitivity of 93% and a specificity of 87% [245].

## CONCLUSIONS

### Bladder Carcinoma is not a “Simple” Pathology

Far from being a *simple* model of tumor, recent studies on BC revealed that several genes and metabolic pathways [147] are involved in its formation and development, and that these alterations are critical for the final outcome. Alterations in gene function may be found at any level – chromosome loss and/or aberrations, point mutations in coding sequences, pre- or post- transcriptional up- and down-regulations, environmental exposure of healthy tissues. As it happens for many tumors, some genes (RAS, TP53, FGFR3) are statistically more frequently altered than others, but in any case it is extremely rare to find any BC sample showing just one genetic lesion. In this perspective, BC – like all malignancies – is a multi-step disease and, as such, tens of cellular malfunctions should be present to induce neoplastic transformation and subsequent malignancy survival. Thus, the original view of BC as a model tumor whose diagnosis and prognosis may be referred to just two main metabolic pathways – controlled by TP53 and FGFR3, respectively – has radically changed in the last few years.

### What Needs to be Done for Bladder Carcinoma?

For many features (for example, the involvement of some key genes such as *TP53* and *FGFR3* that are mutated/altered in many other cancers, or the influence of carcinogenic compounds in its formation), BC is not really different from other tumors. This means that prevention should be a priority in its management. As described in the Introduction, several non-genetic factors affect its epidemiology. Most of them (smoke, professional exposure, diet) might be controlled with a minimal economic effort and, hopefully, reduce its incidence as a consequence. This would greatly help saving lives, improving the life quality of affected patients and, last but not least, also free some additional economical resources that may be used for other medical purposes. Of course, prevention is not the only way to handle this pathology. Genetic predisposition is still cause of a high number of new cases. In this perspective, the greater the knowledge of the biological basis of BC, the higher is the possibility to tailor a therapy according to patients’ characteristics. For example, knowing that the patient with a non-invasive disease has pro-angiogenetic mutations might help in selecting a specific treatment and monitoring this aspect of the disease. But only a profound understanding of the molecular mechanisms underlying this pathology will likely allow obtaining a better and patient-oriented diagnosis and a more efficient handling of BC. The number and quality of molecular studies, and especially the genome-wide oriented ones, are the right way to proceed.

## Figures and Tables

**Table 1.** 2009 Tumor-Nodes-Metastasis (TNM) Classification of Urinary Bladder Cancer [50]

T	Primary Tumor
TX	Primary Tumor cannot be Assessed
T0	No Evidence of Primary Tumor
Ta	Non-invasive Papillary Carcinoma
Tis	Carcinoma *in situ* (‘Flat’ Tumor)
T1	Tumor invades Sub-epithelial Connective Tissue
T2	Tumor Invades Muscle
T2a	Tumor invades Superficial Muscle (Inner Half)
T2b	Tumor invades Deep Muscle (Outer Half)
T3	Tumor invades Perivesical Tissue
T3a	Microscopically
T3b	Macroscopically (Extravesical Mass)
T4	Tumor invades any of the following: Prostate, Uterus, Vagina, Pelvic Wall, Abdominal Wall
T4a	Tumor invades Prostate, Uterus or Vagina
T4b	Tumor invades Pelvic Wall or Abdominal Wall
**N**	**Lymph Nodes**
NX	Regional Lymph Nodes cannot be Assessed
N0	No Regional Lymph Node Metastasis
N1	Metastasis in a Single Lymph Node in the True Pelvis (Hypogastric, Obturator, External Iliac or Presacral)
N2	Metastasis in Multiple Lymph Nodes in the True Pelvis (Hypogastric, Obturator, External Iliac or Presacral)
N3	Metastasis in a Common Iliac Lymph Node(s)
**M**	**Distant Metastasis**
MX	Distant Metastasis cannot be Assessed
M0	No Distant Metastasis
M1	Distant Metastasis

**Table 2.** Metastases of Invasive BC According to [54]

Place	Frequency (%)
Lymph Nodes	78
Liver	38
Lung	36
Bone	27
Adrenal Gland	21
Intestine	13
Heart, Brain, Kidney, Spleen, Pancreas, Meninges, Uterus, Ovary, Prostate, Testes	1-8

**Table 3.** Potential Biomarkers of BC – Gene Functions and Metabolic Pathways According to Genecards (http://www.genecards.org/) Accessed June 2012. For Each Section, Genes are Listed in Alphabetical Order, According to their Full Name (Column 2)

Symbol(s)	Full Name	Molecular Function	Metabolic Pathways	Refs
**Genes Controlling Cell Cycle Regulation**				
CCND1, Bcl1	Cyclin D1	Cyclin	Proliferation, G1/S Transition	[88, 164]
Cdk2/4	Cyclin dependent kinase 2 and 4	Ser/Thr cyclin dependent kinases	Proliferation, G1/S Transition	[82, 83]
CCNE1	Cyclin E1	Cyclin	Proliferation, G1/S Transition	[88]
TP21, p21, CIP1, WAF1, CDKN1A	Cyclin-dependent kinase inhibitor 1A	Protein Repressor	Proliferation, G1/S Checkpoint	[81]
TP27, p27, CDKN4, CDKN1B	Cyclin-dependent kinase inhibitor 1B	Protein Repressor	Proliferation, G1/S Checkpoint	[76, 77, 82, 83]
TP14, p14, NK4a, INK4, ARF, TP16, p16, CDKN2A	Cyclin-dependent kinase inhibitor 2A	Protein Repressor	Proliferation	[171, 187-189]
GATA2, NFE1B	GATA binding protein 2	Transcriptional Activator	Cell Proliferation	[183]
E2F3	E2F transcription factor 3	Transcription Factor	Cell Proliferation	[162]
EZH2, ENX1, KMT6	Enhancer of zeste homolog 2	Transcriptional Repressor	Cell Differentiation	[203]
EOMES; TBR2	Eomesodermin	Transcriptional Activator	Embryo Development	[207]
FGFR3	Fibroblast growth factor receptor 3	Tyrosine Kinase	Cell Cycle Control; Angiogenesis	[131, 132, 217]
GDF-9	Growth differentiation factor-9	Growth Factor	Oncosuppressor; Cell Proliferation	[202]
HOXA9	Homeobox A9	Transcription Factor	Cell Differentiation; Morphogenesis	[207]
ID-1, ID1	Inhibitor of DNA binding 1	HLH-Protein	Cell Proliferation and Senescence; Cell Differentiation	[165]
KRT2A/6B/6C/7/8/10/19/20	Keratins 2A/6B/6C/7/8/10/19/20	Intracellular Structure	Cell Activation and Proliferation	[172, 207, 208]
KRTAP13-1, KRTAP19-2, KRTAP20-2	Keratin-associated proteins 13-1, 19-2, 20-2	Intracellular Structure	Cell Activation and Proliferation	[208]
Nkx.28, NKX2-8	NK2 homeobox 8	Unknown	Control of p27, cyclin D1, FOXO3a	[86]
PLK1	Polo-like kinase 1	Ser/Thr protein kinase	Control of Mitosis	[90]
POU4F2	POU class 4 homeobox 2	Transcription Factor	Cell Differentiation (Putative)	[207]
Rb, RB1	Retinoblastoma	Transcription Repressor	Control of G0/G1 Transition	[89]
RARB, HAP, NR1B2	Retinoic acid receptor b2	Hormone Receptor	Cell Differentiation	[173]
RUNX3, AML2, CBFA3	Runt-related transcription factor 3	Transcription Factor	Activation and Repression of Transcription	[193, 196]
SOX9, CMD1	Sex determining region Y-box 9	Transcription Factor	Chondrogenesis	[194]
TP53, p53	Tumor protein p53	Transcription Factor	Proliferation, Apoptosis, Angiogenesis	[73, 76-78]
TP63, p63	Tumor protein p63	Transcriptional Activator/Repressor	Proliferation, Apoptosis	[75]
TWIST, TWIST1, ACS3, BPES2	Twist homolog 1	Transcription Factor	Cell Differentiation	[214]
TBX2	T-box 2	Unknown	Cell Differentiation	[183]
TBX3	T-box 3	Transcriptional Repressor	Cell Differentiation	[183]
AKT1, AKT, RAC	v-akt murine thymoma viral oncogene homolog 1	Ser-Thr protein kinase	Cell Proliferation; Cell Survival; Angiogenesis	[137, 138, 166]
c-myc, MYC	v-myc myelocystomatosis viral oncogene homolog	Regulation of Gene Transcription	Cell Proliferation	[161]
**Genes Controlling Apoptosis**				
cIAP1, BIRC2, RNF48	Baculoviral IAP repeat containing 2	Protein Inhibitor	Inhibition of Apoptosis	[99]
BIRC5, Survivin	Baculoviral IAP repeat containing 5	Protein Inhibitor	Inhibition of Apoptosis; Cell invasion; Regulator of Mitosis	[96-98]
Bcl-2, PPP1R50	B-cell CLL/lymphoma 2	Control of Mitochondrial Membrane Permeability	Inhibition of Apoptosis	[92, 215]
BNIP3	BCL2/adenovirus E1B interacting protein 3	Calcium Repartitioning	Cell Survival	[116]
c-FLIP, CFLAR	CASP8 and FADD-like apoptosis regulator	Protein Inhibitor	Apoptosis Resistance	[100]
CASP3, CPP32, Yama	Caspase-3	Cysteine-Aspartic Protein Peptidase	Activation of Apoptosis	[92, 93]
DAPK	Death Associated Protein kinase 1	Serine-Threonine Kinase	Apoptosis; Cell Survival	[177, 188, 215, 216]
CD95L, FASLG	Fas ligand TNF superfamily 6	Ligand for Fas	Activation of Apoptosis	[94, 100]
FOXO3a, AF6q21	Forkhead box 3A	Transcription Factor	Activation of Apoptosis	[86]
GDF15, MIC1, PLAB	Growth Differentiation factor 15	Unknown	Activation of Apoptosis	[206]
PMF1	Polyamine-Modulated Factor 1	Polyamine Homeostasis	Control of Cell Growth and Death	[212]
RASSF1A	Ras association domain family member 1	Protein Inhibitor	Cell Proliferation Inhibitor	[187, 189-191, 215]
TMEFF2, tomoregulin-2	Transmembrane protein with EGF-like and follistatin-like domains 2	Activator of Phosphorilation	Cell Survival	[206]
DR4, TNFRSF10A	Tumor necrosis factor receptor superfamily 10A	Death Receptor	Activation of Apoptosis	[101]
DR5, TNFRSF10B	Tumor necrosis factor receptor superfamily 10B	Death Receptor	Activation of Apoptosis	[101]
TRAIL, TNFRSF10C	Tumor necrosis factor receptor superfamily 10C	Protein Inhibitor	Apoptosis Resistance	[101]
Fas, APT1, TNFRSF6	Tumor necrosis factor receptor superfamily 6	Death Receptor	Activation of Apoptosis	[94, 95, 205]
DR3, TNFRSF25	Tumor necrosis factor receptor superfamily member 25	Death Receptor	Activation of Apoptosis	[205]
**Genes Controlling Angiogenesis**				
bFGF, FGF2	Basic fibroblast growth factor	Heparin Binding	Pro-Angiogenesis; Mitogen	[102]
EDNRB	Endothelin receptor type B	G-protein-coupled Receptor	Regulation of Angiogenesis	[204]
Prolidase, PEPD	Peptidase D	Metalloproteinase	Collagen Metabolism; Pro-angiogenesis	[169]
THBS1, TSP1	Thrombospondin-1	Adhesive Glicoprotein; Heparin Binding	Inhibitor of Angiogenesis	[102]
VEGF	Vascular Endothelial Growth Factor	Signaling Protein	Cell Replication and Migration; Inhibition of Apoptosis	[103, 104]
**Genes Controlling Cell-Cell Interactions**				
ADAM12	ADAM metallopeptidase Domain 12	Metalloproteinase	Multinucleate Cell Formation	[127]
ADAM17	ADAM metallopeptidase Domain 17	Metalloproteinase	Release of Cell Surface Proteins	[127]
ADAM28	ADAM metallopeptidase Domain 28	Metalloproteinase	Cell Adhesion	[126]
APC	Adenomatous Polyposis Coli	Antagonist of Wnt Pathway	Cell Migration and Adhesion; Apoptosis	[191, 204]
AR, DHTR, SBMA, AIS	Androgen Receptor	Steroid Hormone Receptor	Cell Growth, Differentiation and Function	[129]
Annexin10, ANXA10	Annexin A10	Unknown	Cell Migration	[121, 122]
APOE, LPG	Apolipoprotein E	Catabolism of Lipoproteins	Cell Function	[130]
BLCA-4	Bladder Cancer A4	Transcription Factor	Metastasis Formation	[167, 168]
BAMBI	BMP and activin membrane-bound inhibitor homolog	Signal Receptor	Metastasis invasion; Cell Movement	[201]
LASS2	Ceramide synthase 2	Sphingolipid Synthesis (Putative)	Metastasis Suppressor	[123]
COL1A2	Collagen Type 1a2	Collagen	Extracellular Matrix Formation	[219]
CTTN, EMS1	Cortactin	Unknown	Cytoskeletal Organization; Regulation of Cell-Cell Junctions; Regulation of Invasiveness	[124]
CDH1	E-cadherin	Calcium-Dependent Membrane Protein	Cell Adhesion	[128, 129, 184]
ERa, ESR1	Estrogen Receptor Alpha	Steroid Hormone Receptor	Cell Growth, Differentiation and Function	[129]
FGB	Fibrinogen Beta Chain Precursor	Polymerization of Monomers	Cell Adhesion	[130]
HYAL-1, LUCA1, NAT6	Hyaluronoglucosaminidase 1	Degradation of Hyaluronic Acid	Cell Proliferation, Migration and Differentiation	[218]
MAPK	Mitogen-activated protein kinase family	Ser-Thr kinase	Cell Growth, Adhesion, Survival and Differentiation	[137, 138]
NID2	Nidogen2	Membrane Glycoprotein	Cell Adhesion	[214]
PTEN, BZS, MMAC1,	Phosphatase and tensin honolog	Protein and Lipid Phosphatase	Cell Migration Inhibition	[178]
PIK3CA	Phosphoinositide-3-kinase catalytic alpha polypeptide	Lipid Kinase	Cell Growth, Survival, Proliferation, Motility and Morphology	[119, 137, 138]
PLCG1	Phospholipase C, g1	Phospholipase	Actin Organization; Cell Migration	[137, 138]
PFN1	Profilin-1	Actin Binding	Cytoskeletal Organization	[125]
PRKCI	Protein kinase C	Ser-Thr kinase	Modulation of Membrane Structure	[137, 138]
RIN1	Ras and Rab interactor 1	Ras Effector Protein	Cytoskeletal Remodeling	[115]
RAS	Rat sarcoma viral oncogene family	GTPase	Activation of Mitosis	[114]
SFRP	Secreted frizzled receptor protein family	Protein Receptor	Metastasis invasion; Cell Movement	[199, 200]
SERPINA1	Serpin peptidase inhibitor 1	Alpha-1 Antitrypsin	Inhibition of Elastase	[130]
STAT1, STAT91	Signal transducer and activator of transcription 1	Signal Transducer	Response to Growth Factors	[139]
TIMP-3	TIMP metallopeptidase inhibitor 3	Proteinase	Cell Remodeling	[213]
TSC1, LAM, hamartin	Tuberous sclerosis 1	Protein Inhibitor	Inhibition of Nutrient-Mediated Cell Growth	[120]
UPK	Uroplakin	Integral Membrane Proteins	Cytoskeleton Regulation	[107]
HER2, ERBB2	v-erb-b2 erythroblastic leukemia viral oncogene homolog 2	Epidermal Growth Factor Receptor; Tyrosine Kinase	Activation of Mitosis	[108, 109]
Src, ASV	v-src sarcoma viral oncogene homolog	Tyrosine Kinase	Cell Proliferation, Survival and Migration	[137, 138]
**Other Genes**				
hOGG1	8-oxoguanine DNA glycosilase	Glycosylase	DNA Base Excision Repair	[142]
C10orf116	Chromosome 10 open reading frame 116	Unknown	Unknown	[172]
CYP1B1	Cytochrome P450 1B1	Oxidation	Chemical Modification of Various Compounds; Detoxification	[151]
ERCC1	Excision repair cross-complementing rodent repair deficiency, group 1	Endonuclease	DNA Nucleotide Excision Repair	[143]
ERCC2, XPD, TFIIH	Excision repair cross-complementing rodent repair deficiency, group 2	Helicase	Double-Stranded DNA Breaks Repair	[142, 144]
GSTM1	Glutathione S-transferase mu 1	Chemical Conjugation	Detoxification	[149, 150]
GSTP1	Glutathione S-transferase pi 1	Chemical Conjugation	Detoxification	[149, 150]
GSTT1	Glutathione S-transferase theta 1	Chemical Conjugation	Detoxification	[149, 150]
HMGB1, SBP1	High mobility group box 1	DNA Bending	DNA Transcription and Repair	[140]
SIRT2	Histone Deacetylase	Chromatin Remodeling	Gene Expression Control	[124]
HDAC6	Histone Deacetylase 6	Chromatin Remodeling	Gene Expression Control	[124]
LRG1	Leucine-rich alpha-2-glycoprotein 1	Unknown	Unknown	[130]
MTHFR	Methylenetetrahydrofolate reductase NAD(P)H	Reductase	Nucleotide Biosynthesis	[141]
NBS1, NBN	Nibrin	DNA Damage Signaling	Double-Stranded DNA Breaks Repair	[144]
PARP1, PPOL	Poly(ADP-ribose) polymerase 1	DNA Polymerase	Base Excision DNA Repair	[146]
SYNPO2, Myopodin	Synaptopodyn 2	Actin-binding Protein	Unknown	[211]
UGT	UDP glucuronosyltransferase family	Chemical Modification of xenobiotics	Detoxification	[148]
VIM	Vimentin	Type III Intermediate Filament	Cell Structure	[206]
XPC, Rad4, P125	Xeroderma pigmentosum, group C	DNA Bending	Nucleotide Excision DNA Repair	[144]
XRCC3	X-ray repair complementing defective repair in Chinese hamster cells 3	Unknown	Double-Stranded DNA Breaks Repair by Homologous Recombination	[145]
ZIC4	Zic family member 4	DNA Binding	Unknown	[183]
ZNF154	Zinc finger protein 154	Transcriptional Regulation (Putative)	Unknown	[191]

**Table 4.** Record of Non Coding RNAs (ncRNA) Cited in the Text that are Important in BC Formation and/or Development

ncRNA	Target(s)	Reference(s)
miR1	SRSF9/SRp30c	[244]
miR100	FGFR3	[235]
miR125b	E2F3	[242, 243]
miR129	GALNT1	[259]
	SOX4	
miR143	ERK5/MAPK7	[254, 255]
	AKT	
miR1826	CTNNB1	[253]
	MEK1	
	VECFG	
miR195	CDK-4	[246]
miR200	ERRF-1	[247]
	ZEB1	[248-250]
miR205	TP53	[245]
	PTEN	
	c-erbB-3	
	cdc42	
	Yes	
mir21	TP53	[228, 234]
	TIMP3	[236]
	Bcl-2	
	PTEN	[237, 238]
	TPM1	[239]
	MSH2	[240]
	E2F3	[162, 241]
miR221	TRAIL	[257, 258]
miR31	FGFR3	[228]
mir449a	CDK6	[251]
	CDC25a	
	TP130	
miR493	FZD4	[252]
	RhoC	
miR99a	FGFR3	[234]
UCA1	CREB	[222]

Note that most of the Listed Target Genes are also Present in Table 3, Indicating that the Same Gene Might be Involved in this Pathology Either Because of Mutations in the Coding Sequence or Because of its Mis-Regulation.
